# Utilizing Balance Assessment Tools by Physical Therapists for Patients with Balance Disorders

**DOI:** 10.3390/healthcare13080928

**Published:** 2025-04-17

**Authors:** Abdulaziz A. Albalwi, Ahmad A. Alharbi, Hamad S. Al Amer, Samia A. Alamrani, Hani F. Albalawi, Maryam K. Alatawi, Wahaj A. Albalawi, Basmah A. Albalawi, Amani M. Qari, Waad A. Alatawi, Jamsheed Javid

**Affiliations:** 1 Department of Health Rehabilitation Sciences, Faculty of Applied Medical Sciences, University of Tabuk, Tabuk 71491, Saudi Arabia; aaalharbi@ut.edu.sa (A.A.A.); halamer@ut.edu.sa (H.S.A.A.); salamrani@ut.edu.sa (S.A.A.); hf_albalawi@ut.edu.sa (H.F.A.); 421000999@stu.ut.edu.sa (M.K.A.); 421000722@stu.ut.edu.sa (W.A.A.); 421000144@stu.ut.edu.sa (B.A.A.); 421003118@stu.ut.edu.sa (A.M.Q.); 421001059@stu.ut.edu.sa (W.A.A.); 2 Department of Medical Laboratory Technology, Faculty of Applied Medical Sciences, University of Tabuk, Tabuk 71491, Saudi Arabia; jali@ut.edu.sa

**Keywords:** postural stability, balance, balance disorders, physical therapists, assessment, physical rehabilitation

## Abstract

**Background/Objectives:** Physical therapists’ use of various balance assessment tools is essential for accurately identifying deficits and guiding rehabilitation plans. This study aimed to investigate clinical balance assessment practices in Saudi Arabia, examine physical therapists’ preferences for different balance assessment tools, and analyze how participant characteristics—such as age, experience, and practice setting—affect these preferences. **Methods:** A descriptive cross-sectional study was conducted between April and July 2024 in Saudi Arabia. A total of 194 physical therapists (62.9% male; 45.9% with 1–5 years of experience) who actively manage individuals with balance disorders were recruited using a convenience sampling technique. Data were collected through a self-structured questionnaire. Participants reported their use of balance assessment tools on a six-point Likert scale, incorporating both numeric and descriptive anchors. **Results:** The Single-Leg Stance was the most regularly used tool (54.6%), followed by the Berg Balance Scale and Functional Gait Assessment (FGA) (both 43.8%). Conversely, tools such as the Performance-Oriented Mobility Assessment (68.1%), Mini Balance Evaluation System (65.4%), and Fall Efficacy Scale International (56.2%) were the most underutilized. Significant associations were observed between tool preferences and participant characteristics, including area of practice, academic qualification, experience level, and work environment (*p* < 0.05). However, several validated assessment tools remain underutilized, highlighting gaps in awareness and training. **Conclusions:** The findings of this study highlight the need for greater standardization in balance assessment practices. Improving training programs, establishing clear clinical guidelines, and standardizing assessment protocols across healthcare settings can help make balance evaluations more consistent and effective.

## 1. Introduction

Balance control is a critical function that enables individuals to maintain stability during both stationary and dynamic activities [1]. It is essential for performing various functional tasks, including walking, standing, reaching, and transitioning between positions [2]. Body balance is defined as the ability to maintain the body’s center of gravity within the base of support [1]. Globally, balance impairments are prevalent, affecting up to 26.5% of older people [3]. In Saudi Arabia, studies estimate that more than 25%, even up to 49.9%, of adults aged 65 and older experience balance impairments [4,5]. This prevalence increases further with age and the presence of comorbidities such as stroke, diabetes, and arthritis [5].

Impaired body balance can result from various conditions, including musculoskeletal abnormalities, proprioceptive and somatosensory dysfunction, central or peripheral nervous system injuries, and age-related changes [6]. Because balance plays a crucial role in overall mobility and well-being, its impairment can significantly reduce quality of life and increase the risk of falls and injuries [7].

Balance impairments pose significant physical, psychological, and social challenges for older adults [8,9,10]. Physically, they increase the risk of falls, which are a leading cause of injuries, hospitalization, and disability. Falls can also result in long-term mobility issues and the loss of independence. Psychologically, the fear of falling reduces confidence, increases anxiety, and leads to activity avoidance, creating a cycle of inactivity and further physical decline [9]. Socially, this fear often causes isolation and reduced participation in activities, impacting mental well-being and quality of life [10]. Early assessment and intervention are essential to break this cycle, improve mobility, and enhance overall health in older adults.

Assessing balance is challenging, as effective balance control depends on the coordinated interaction of multiple body systems, including the vestibular, visual, somatosensory, and neuromuscular systems [11]. These systems work together to maintain equilibrium by supporting static balance, which ensures stability in stationary positions, and dynamic balance, which sustains stability during movement and anticipatory adjustments [2]. Consequently, comprehensive balance assessment must account for all these interconnected systems to accurately identify impairments and guide effective interventions [2,11,12]. Given the importance of accurate assessment, physical therapists play a vital role in evaluating and rehabilitating individuals with balance disorders. Using valid, reliable, and standardized assessment tools is essential for ensuring effective treatment and improving patient outcomes. Clinical balance tests are widely used to assess different aspects of balance control, helping physical therapists tailor interventions to each patient’s specific needs.

Numerous valid and reliable balance tests are currently available [12,13,14,15]. These tests primarily assess functional performance by observing and evaluating specific quantifiable parameters [12,13,14,15]. However, they vary in terms of the populations for which they have been validated and the specific aspects of balance they assess, such as static stability, dynamic control, or reactive and anticipatory responses [13,14,15]. This variability complicates direct comparisons between different measures. Therefore, current clinical guidelines recommend using standardized balance assessment tests.

While several review articles have suggested standardized balance measures to guide clinicians in selecting appropriate tests, few studies have explored current clinical practices [15,16,17]. A study conducted in Ontario revealed significant differences among practice groups [15]. For instance, 43.4% of respondents found current standardized methods to be satisfactory, while 79% expressed a desire to improve their assessment approaches by addressing individual, contextual, and measure-specific barriers. One of the most common barriers reported in this study was a deficiency in time and knowledge [15].

Despite existing research on balance assessment, no studies have specifically examined the practices of Saudi physical therapists in evaluating balance impairments. According to the biopsychosocial health model, physical therapists must consider not only the biomechanical and neurological aspects of balance but also the psychosocial factors that could influence patients’ ability to maintain stability [18]. Therefore, this study aimed to explore the clinical practices of Saudi physical therapists in balance assessment across various healthcare settings; assess their preferences for different balance assessment tools; and analyze how factors such as age, experience, and practice setting influence these choices.

By identifying the most frequently used tools and the factors that affect their selection, this study is anticipated to provide valuable insights to inform training programs, ensuring that physical therapists are well equipped with the necessary skills to accurately assess balance. Additionally, the findings could guide future research in developing or refining balance assessment tools tailored to the unique needs of the Saudi population, ultimately improving patient care and reducing the risk of falls and related injuries.

## 2. Materials and Methods

### 2.1. Study Design and Ethical Considerations

A descriptive cross-sectional study was conducted among licensed physical therapists working as clinicians in hospitals and clinics across various regions of the Kingdom of Saudi Arabia from April to July 2024. This study complied with all relevant institutional policies and adhered to the principles of the Declaration of Helsinki. Ethical approval was obtained from the Local Research Ethics Committee (LREC) at the University of Tabuk (UT-382-206-2024). Informed consent was obtained from all participants before participation.

### 2.2. Participants

There were almost 7000 physical therapists in Saudi Arabia in 2023 [19]. The sample size calculation was performed using G-power with a power of 80%, a margin error of 5%, and a medium effect size of 0.3. Therefore, the sample size required was 365 physical therapists. The target population included physical therapists actively practicing in the neurological, vestibular, geriatric, pediatric, sports, and orthopedic domains. Physical therapists who were not involved in the assessment and treatment of individuals with balance disorders, as well as those exclusively in academia or still in training, including interns or students, were excluded due to their limited direct patient contact and exposure in clinical settings. Eligible participants included both male and female physical therapists with at least a bachelor’s degree in physical therapy and a minimum of one year of clinical experience [20].

### 2.3. Measures

#### Questionnaire

A self-structured questionnaire was developed with help from previous studies [15,21,22] to investigate how physical therapists in Saudi Arabia use balance assessment tools in clinical practice. It incorporated 14 questions, starting with 2 questions regarding a consent and screening question, where participants were asked if they agreed to participate in this study and whether they assessed and treated patients with balance. After that, the participants completed the following three parts of the questionnaire: (1) demographics, which contained 6 questions related to the participant’s gender, age group, region of practice within Saudi Arabia, year of graduation, years of clinical experience, and highest degree obtained in physical therapy; (2) general practice when assessing and treating patients with balance disorders, which included 5 questions about the number of years they have been treating balance disorders, the age range of patients they work with, the type of facility they work in (e.g., government, private, or university), their work environment (individual, with other physical therapists, or as part of a multidisciplinary team), and their primary area of clinical practice (e.g., vestibular rehabilitation, neurology, orthopedics); and (3) balance assessment tools and outcome measures available at the practice. The participants were asked to answer a question to rate their frequency of use for a range of standardized balance assessment tools, including 16 components: the Activity-Specific Balance Confidence Scale (ABC), Berg Balance Scale (BBS), Fall Efficacy Scale International (FES-I), Dynamic Gait Index (DGI), Functional Reach Test, Single-Leg Stance (SLS), Four-Step Square Test, Y-Balance Test (Lower Quarter), Mini Balance Evaluation Systems Test, Performance-Oriented Mobility Assessment (POMA—Tinetti), Turn Test 360°, Functional Gait Assessment, Trunk Impairment Scale, Postural Assessment Scale for Stroke Patients, Timed Up and Go (TUG), and Five Times Sit-to-Stand. Responses were recorded using a 6-point Likert scale with both numeric and descriptive anchors, as follows: “Usually” (>80%), “Mostly” (41–79%), “Occasionally” (21–40%), “Sometimes” (1–20%), “Never” (0%), and “Not Familiar at All”.

The questionnaire underwent a pilot testing and content validity phase with the assistance of a convenience sample of 10 physical therapists working in various practice settings. They were asked to evaluate whether the scale items were clear, relevant, and representative of the construct being measured. All physical therapists agreed that the scale was appropriate and comprehensive, and no significant concerns or modifications were suggested. The reliability of the questionnaire was assessed using Cronbach’s α to evaluate internal consistency. A Cronbach’s alpha value of 0.891 was obtained, indicating good internal consistency [23].

### 2.4. Procedure

**Figure 1:**
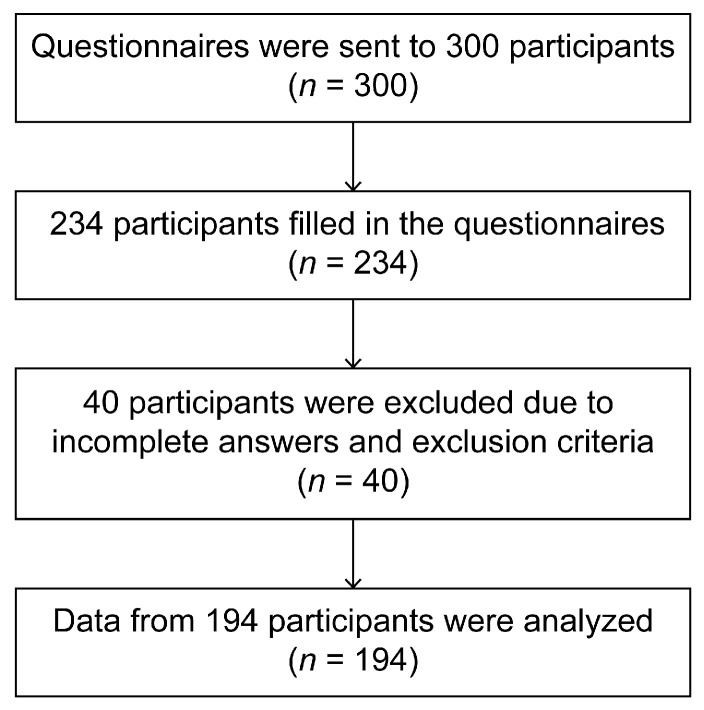
Flowchart on participants’ responses.

Data were collected using an electronic questionnaire distributed via Google Forms to maximize participant reach across Saudi Arabia. The questionnaire included an informed consent statement on the first page, requiring participants to indicate their agreement to participate by checking a consent box. Data collection took place between April 2024 and July 2024 using a convenience sampling technique to enhance participation. The questionnaire was sent via email to approximately 300 physical therapists across the Kingdom of Saudi Arabia, yielding 234 responses (78%). After reviewing the responses, 40 incomplete or ineligible responses were excluded based on the predefined exclusion criteria. Consequently, 194 completed questionnaires were analyzed using SPSS version 25 (IBM Corp, Armonk, NY, USA), as shown in Figure 1.

### 2.5. Statistical Analyses

Demographic and work-related data, including age, sex, practicing region in Saudi Arabia, years of experience, year of graduation, primary area of practice, work environment, and the age range of patients assessed and treated, were presented in frequencies and percentages. Likert-scale responses were grouped into the following three categories: “Never” (combining “Not Familiar at All” and “Never”), “Occasionally” (combining “Sometimes” and “Occasionally”), and “Regularly” (combining “Mostly” and “Usually”).

Statistical analyses assessed participants’ ratings of balance assessment tools and the impact of work-related characteristics on tool preference. To compare the participants’ ratings for balance assessment tools, significant differences in ratings were examined, followed by follow-up comparisons when applicable. To examine the impact of participant characteristics on tool preferences, the proportions of participants who reported regular use of each scale were correlated with work-related characteristics. Because all variables were categorial (nominal and ordinal), normality testing was not performed. Non-parametric tests (Chi-square tests) were used to determine statistical significance, which do not require the assumption of normality. All analyses were conducted using SPSS for Windows version 25.0 with an alpha level set at 0.05. The effect size (ES) for the observed differences was estimated using Cohen’s ω and was interpreted as follows: values between 0.10 and 0.30 indicated a small ES, values between 0.30 and 0.50 indicated a medium ES, and values of 0.50 or above indicated a large ES [24].

## 3. Results

### 3.1. Participants’ Characteristics

Table 1 shows the characteristics of the study participants. Out of 194 physical therapists, 62.9% were male and 37.1% were female. Most of the respondents had a bachelor’s degree (65.5%), while 21.6% had a master’s degree, 8.8% had a doctoral degree, and only 3.1% were Doctors of Physical Therapy. In terms of years of experience, 45.9% had up to 5 years of experience, 24.7% had less than 1 year of experience, and 16% had more than 11 years of experience. Nearly half of the participants (49%) reported working at governmental hospitals/clinics, and the others (45.4%) reported working at private hospitals/clinics. Approximately 40% of the participants’ primary areas of practice were neurological, 16% were sports, 14% were orthopedic, 13% were vestibular rehabilitation, 10% were pediatric, 3.6% were geriatric, and only 1% were cardiopulmonary rehabilitation. Among the participants in this study, 38.7% were working individually, 26.3% were attached to other physical therapists, and 35.1% were part of a multidisciplinary team. A total of 71 (36.6%) respondents were practicing in the middle region, 32% in the western region, and the minimum representation was from the eastern region (8.8%). In terms of the patient age range assessed and treated, 36.1% treated early-aged adults, 30.9% treated middle-aged adults, 19.1% treated children, and 13.9% treated older adults.

### 3.2. Balance Assessment Tool Ratings

**Figure 2:**
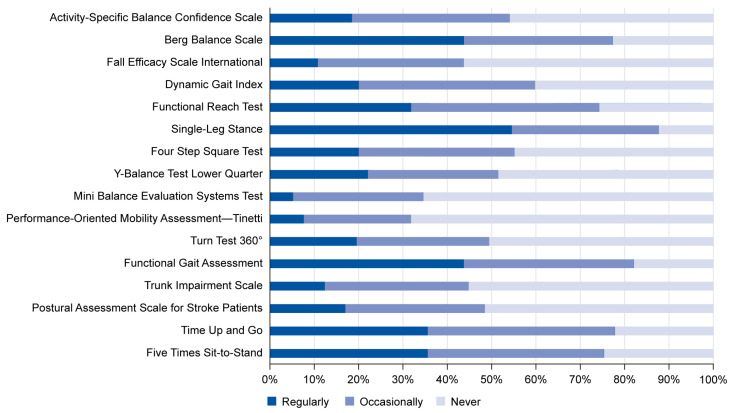
Participants’ ratings for balance assessment tools. The figure shows that the Single-Leg Stance test was the most regularly used tool among physical therapists (54.6%), followed by the Berg Balance Scale, Functional Gait Assessment, and Timed Up and Go test. Several tools, including the POMA—Tinetti, Mini Balance Evaluation Systems Test, and Fall Efficacy Scale International, had high rates of non-use, reflecting limited familiarity or perceived applicability in clinical settings.

Figure 2 illustrates the participants’ ratings for balance assessment tools. The analysis revealed that the Single-Leg Stance (SLS) is the most regularly used tool (54.6%). Furthermore, the percentages of the participants who reported that they regularly and occasionally use the Berg Balance Scale (BBS), Functional Gait Assessment (FGA), and Timed Up and Go (TUG) were equally significant. The Functional Reach Test (FRT) (42.3%) and Five Times Sit-to-Stand (STS) (39.7%) were reported to be occasionally used by a significant number of the sample. In contrast, significant proportions of the participants reported that they have never used the Performance-Oriented Mobility Assessment (POMA—Tinetti) (68.1%), the Mini Balance Evaluation System (MBEST) (65.4%), the Fall Efficacy Scale International (FES-I) (56.2%), the Trunk Impairment Scale (TIS) (55.2%), the Turn Test 360° (50.5%), or the Postural Assessment Scale for Stroke Patients (PASSP) (51.5%). Additionally, the proportions of participants who reported that they never and occasionally use the Activity-Specific Balance Confidence Scale (ABC), Dynamic Gait Index (DGI), and Four-Step Square Test (FSST) were equally significant. Table 2 shows detailed results of the statistical analyses performed regarding the participants’ ratings for balance assessment tools.

### 3.3. Effect of Participant Characteristics on Tool Preferences

The results revealed significant relationships between participant work-related characteristics and the BBS, SLS, FGA, and TUG. As shown in Table 3 and Table 4, a significant number of participants, for whom their primary area of practice was neurological, reported that they regularly used the BBS (48.1%, *p* = 0.007, ES = 0.36), SLS (54.4%, *p* < 0.005, ES = 0.53), FGA (44.3%, *p* = 0.003, ES = 0.39), and TUG (43.0%, *p* = 0.001, ES = 0.41). Most of those who worked in orthopedics reported regular employment of the SLS (71.4%, *p* < 0.005, ES = 0.81) and FGA (57.1%, *p* = 0.025, ES = 0.51). Furthermore, significant proportions of pediatric physical therapists stated their consistent use of the BBS (57.1%, *p* = 0.028, ES = 0.58) and SLS (71.4%, *p* = 0.001, ES = 0.82). Finally, the majority of physical therapists who practiced in the field of sports described the SLS (53.1%, *p* = 0.033, ES = 0.46) as the tool they used on a regular basis. Regarding the effect of academic degree on tool preferences, a significant percentage of respondents with a bachelor’s degree selected the SLS (55.9%, *p* < 0.005, ES = 0.50) and FGA (44.1%, *p* = 0.001, ES = 0.33) as the tools they regularly use. A substantial number of participants who held master’s degrees in physical therapy were found to utilize the BBS (59.5%, *p* = 0.001, ES = 0.57), SLS (52.4%, *p* < 0.005, ES = 0.62), and TUG (45.2%, *p* = 0.005, ES = 0.51) more regularly. Lastly, most doctoral degree holders specified the BBS (64.7%, *p* = 0.011, ES = 0.73) as the tool of choice for evaluating patients with balance disorders.

In terms of participants’ experience, the majority of physical therapists with 1 to 5 years of practicing experience stated their consistent use of the BBS (43.8%, *p* = 0.033, ES = 0.28), SLS (59.6%, *p* < 0.005, ES = 0.61), and FGA (49.4%, *p* < 0.005, ES = 0.41). Similarly, the BBS (61.3%, *p* = 0.004, ES = 0.60), SLS (61.3%, *p* = 0.003, ES = 0.61), and TUG (45.2%, *p* = 0.020, ES = 0.50) were found to be regularly used by those with 11 years of experience or more. The results also revealed that most of the participants who were treating children and adolescents utilized the BBS (51.4%, *p* = 0.032, ES = 0.43) and SLS (62.2%, *p* < 0.005, ES = 0.64) as the primary tool for evaluating patients with balance disorders, while most of those who work with early-aged adults stated their primary use of the SLS (52.9%, *p* < 0.005, ES = 0.47). Additionally, significant proportions of physical therapists who were dealing with middle-aged adults reported that the SLS (56.7%, *p* < 0.005, ES = 0.57), FGA (46.7%, *p* = 0.008, ES = 0.40), and TUG (45.0%, *p* = 0.019, ES = 0.36) were the most regularly used tools.

The BBS (50.5%, *p* < 0.005, ES = 0.43), SLS (55.8%, *p* < 0.005, ES = 0.52), and FGA (44.2%, *p* = 0.005, ES = 0.34) were identified as the main tools used by most physical therapists who worked in public hospitals. Moreover, the majority of private hospitals’ physical therapists selected the SLS (52.3%, *p* < 0.005, ES = 0.49) and FGA (43.2%, *p* = 0.009, ES = 0.33) as their regularly used tools, while most of those who were working at university hospitals reported the BBS (72.7%, *p* = 0.020, ES = 0.84).

Working environment was also significantly correlated with tool preferences. It was found that a significant proportion of physical therapists who were working individually reported the use of the SLS (53.3%, *p* < 0.005, ES = 0.47) on a regular basis at their practice, while the majority of those who were attached to other physical therapists chose the SLS (54.9%, *p* = 0.001, ES = 0.51) and FGA (43.1%, *p* = 0.012, ES = 0.42). Most of the physical therapists that were part of a multidisciplinary team stated the BBS (48.5%, *p* = 0.001, ES = 0.47), SLS (55.9%, *p* < 0.005, ES = 0.58), and FGA (50.0%, *p* = 0.002, ES = 0.43) as the primary tools they use. Finally, in this study, no significant preferences were identified among the respondents for the ABC, FES-I, DGI, FRT, FSST, Y-Balance, MBEST, POMA—Tinetti, Turn Test 360°, TIS, PASSP, and ST.

## 4. Discussion

This study provides valuable insights into the current clinical practices of Saudi Arabia’s physical therapists in assessing balance impairments. The findings highlight significant variations in the selection and utilization of balance assessment tools based on different factors such as primary area of practice, level of education, years of experience, and work setting.

The present study revealed that the SLS test was the most regularly used balance assessment tool (54.6%), followed by the BBS, FGA, and TUG, which were also widely utilized. The SLS is a valid and reliable tool that is commonly used by physical therapists due to its simplicity and efficiency [25,26]. These findings align with the preferences of physical therapists in Canada [21] and Saskatchewan [22], where static balance measures were preferred for their practicality and ease of implementation in neurological and orthopedic settings [15]. A cross-sectional study reported that out of 369 participants, 70% of them reported using the SLS and BBS, supporting their widespread clinical utility. In contrast, another study identified that the BBS was the most commonly used assessment tool for balance among physical therapists [21,27,28], indicating variations in regional preferences and clinical emphasis. The TUG test was perceived as useful by 56.9% of the respondents; however, there were significant differences across the practice groups, suggesting that professional background and specialty influence tool selection [15].

A notable trend in this study is the underutilization of standardized tools, such as the POMA—Tinetti, MBEST, and FES-I. This finding is consistent with international studies, which have reported that limited knowledge, training opportunities, and time constraints often contribute to the infrequent use of these tools [15,29]. The limited use of these tools in Saudi Arabia can be explained by the fact that physical therapists tend to rely on familiar, quick-to-administer assessments, even when more comprehensive tools are available. Therefore, targeted training programs could enhance the adoption of underutilized but valuable tools in clinical practice.

The results of this study suggest that the primary area of practice plays a significant role in determining the preferred balance assessment tool. Neurological and pediatric therapists predominantly used the BBS and SLS, while orthopedic and sports therapists preferred the SLS and FGA. These preferences align with the functional demands of each specialty. For instance, the BBS is commonly used for assessing static and dynamic balance in neurological populations, while the FGA is widely adopted in orthopedic and sports settings to evaluate gait and postural stability.

Our findings further revealed that the BBS is particularly favored in neurological rehabilitation, followed by orthopedic, pediatric, and vestibular rehabilitation. However, physical therapists working in cardiopulmonary settings reported that they never used the BBS in their practice. This suggests that while the BBS is widely accepted in balance assessment, its applicability in cardiopulmonary rehabilitation may be limited or underrecognized. Similar trends have been reported in previous reports, where balance assessments in cardiopulmonary rehabilitation rely more on functional endurance tests than traditional static balance measures [30].

Additionally, academic qualifications and clinical experience influenced tool preferences. Physical therapists with a master’s or doctoral degree were more likely to use standardized tools such as the BBS and TUG, possibly due to their greater exposure to evidence-based practice. Similarly, those with over 11 years of experience favored the BBS and SLS, whereas physical therapists with fewer years of practice relied more on easily administered tools like the SLS. This can be attributed to the fact that clinicians with a higher level of education have acquired more in-depth knowledge and training, which enables them to appreciate the benefits of standardized tools in enhancing diagnostic accuracy and treatment planning [30]. Work environment also influenced tool selection, with physical therapists working in multidisciplinary teams reporting greater use of standardized tools (BBS, SLS, and FGA) compared to those working individually. This may be due to collaborative clinical decision-making and shared knowledge among professionals in team-based settings.

Our study also supports findings from a Canadian study in Saskatchewan [22], where physical therapists regularly used multiple assessment measures, with movement observation being the most common, followed by the BBS, SLS, and tandem standing/walking. This highlights the fact that, while static and functional assessments dominate clinical practice, integrating multiple approaches may provide a more comprehensive evaluation of balance impairments [22].

The findings of this study highlight the importance of establishing more consistent balance assessment practices across Saudi Arabia. While tools like the BBS, SLS, and FGA are commonly used, many other validated measures remain underutilized, pointing to a potential gap in awareness and training. To address these inconsistencies, several steps can be taken to improve clinical application and physical therapists’ training and education. First, the development of national clinical guidelines for the assessment of balance would be a valuable step towards standardization. Such guidelines should recommend specific tools for use in specific clinical settings. Second, specific educational programs for practicing physical therapists can help reduce the knowledge gap and confidence when using underutilized tools like the POMA—Tinetti, MBEST, and FES-I. These programs should include hands-on workshops, case-based learning, and peer discussion sessions to enhance familiarity and competence in applying these assessment tools. Additionally, universities and training institutions should incorporate balance assessment training into physical therapy curricula, with an emphasis on the psychometric properties and clinical applicability of each tool.

Additionally, this study emphasizes how work environments and experience levels influence decision-making in clinical practice. Standardizing assessment protocols across various healthcare settings—especially in private and university hospitals—could help promote greater consistency. Early-career physical therapists may especially benefit from targeted educational and mentorship programs designed to enhance their familiarity with different balance assessment tools. Experienced physical therapists, who are more likely to use a broader range of standardized tools, can serve as mentors to support and guide newer physical therapists in making informed assessment choices. Such initiatives could build a strong culture of evidence-based practice and continuous professional development.

Despite its strengths, this study has some limitations. The sample size and response rate for this study were lower than expected, which may have influenced the overall findings. Additionally, the questionnaire focused on general balance assessment practices without distinguishing between different causes of balance disorders. Future research could improve this by including more specific questions that explore how clinicians assess balance based on the underlying condition and its unique symptoms.

Future research should provide a more in-depth exploration of the challenges preventing the widespread adoption of standardized balance assessments. Factors such as time limitations, insufficient training, and limited access to resources need to be explored in greater detail to develop practical solutions. Additionally, qualitative studies such as focus groups or clinician interviews could provide valuable insights into the reasoning behind tool selection in real-world practice. Expanding this research to include rehabilitation centers and rural healthcare settings would offer a more comprehensive picture of balance assessment practices across different patient populations and healthcare environments.

## 5. Conclusions

This study is the first to provide an in-depth investigation of balance assessment practices among physical therapists in Saudi Arabia, revealing significant variations influenced by work settings, experience levels, and primary areas of practice. While tools such as the SLS, BBS, and FGA are widely used, the limited adoption of other validated measures highlights the need for greater training, awareness, and standardization. By investing in education, refining clinical guidelines, and fostering interdisciplinary collaboration, healthcare providers can enhance the accuracy and effectiveness of balance assessments. Ultimately, these improvements will lead to better patient outcomes, stronger fall prevention strategies, and a higher standard of care for individuals suffering from balance impairments.

## Figures and Tables

**Table 1:** Demographics and characteristics of the respondents in relation to assessing and treating patients with balance disorders.

Characteristic	Number (*n*)	Percentage (%)
Gender		
Male	122	62.9
Female	72	37.1
Age (years)		
20–30	126	64.9
31–40	48	24.7
41–50	16	8.2
51 and more	4	2.1
Qualification (physical therapy)		
Diploma	2	1.0
Bachelor’s	127	65.5
Doctor of Physical Therapy (DPT)	6	3.1
Master’s	42	21.6
Doctorate	17	8.8
Practicing experience		
>1 years	48	24.7
1–5 years	89	45.9
6–10 years	26	13.4
11 years and more	31	16.0
Facility type		
Government hospital/clinic/center	95	49.0
Private hospital/clinic/center	88	45.4
University hospital	11	5.7
Primary area of practice		
Vestibular rehabilitation	25	12.9
Neurological	79	40.7
Sports	32	16.5
Orthopedic	28	14.4
Pediatric	21	10.8
Geriatric	7	3.6
Cardiopulmonary	2	1.0
Women’s health	0.0	
Work environment		
Work individually	75	38.7
Work with other physiotherapists only	51	26.3
Work in a multidisciplinary team (with at least one other type of health professional)	68	35.1
Patient age range assessed and treated		
Children and adolescents (less than 18 years old)	37	19.1
Early-aged adults (18–39 years old)	70	36.1
Middle-aged adults (40–60 years old)	60	30.9
Older adults (61 and more than)	27	13.9
Practicing region (Saudi Arabia)		
Middle region	71	36.6
Western region	62	32.0
Eastern region	17	8.8
Northern region	26	13.4
Southern region	18	9.3

**Table 2:** Main differences and follow-up comparisons of participants’ ratings for balance assessment tools (*n* = 194).

Tool	Never (%)	Occasionally (%)	Regularly (%)	*p*-Value	Pairwise Comparison	*p*-Value
ABC	89 (45.9)	69 (35.6)	36 (18.5)	<0.005 *	Never vs. Occasionally	0.112
					Never vs. Regularly	<0.005 *
					Occasionally vs. Regularly	0.001 *
BBS	44 (22.7)	65 (33.5)	85 (43.8)	0.002 *	Never vs. Occasionally	0.044 *
					Never vs. Regularly	<0.005 *
					Occasionally vs. Regularly	0.102
FES-I	109 (56.2)	64 (33.0)	21 (10.8)	<0.005 *	Never vs. Occasionally	0.001 *
					Never vs. Regularly	<0.005 *
					Occasionally vs. Regularly	<0.005 *
DGI	78 (40.2)	77 (39.7)	39 (20.1)	<0.005 *	Never vs. Occasionally	0.936
					Never vs. Regularly	<0.005 *
					Occasionally vs. Regularly	<0.005 *
FRT	50 (25.8)	82 (42.3)	62 (31.9)	0.018 *	Never vs. Occasionally	0.005 *
					Never vs. Regularly	0.257
					Occasionally vs. Regularly	0.096
SLS	24 (12.4)	64 (33.0)	106 (54.6)	<0.005 *	Never vs. Occasionally	<0.005 *
					Never vs. Regularly	<0.005 *
					Occasionally vs. Regularly	0.001 *
FSST	87 (44.8)	68 (35.1)	39 (20.1)	<0.005 *	Never vs. Occasionally	0.127
					Never vs. Regularly	<0.005 *
					Occasionally vs. Regularly	0.005 *
Y-Balance	94 (48.5)	57 (29.4)	43 (22.1)	<0.005 *	Never vs. Occasionally	0.003 *
					Never vs. Regularly	<0.005 *
					Occasionally vs. Regularly	0.162
MBEST	127 (65.4)	57 (29.4)	10 (5.2)	<0.005 *	Never vs. Occasionally	<0.005 *
					Never vs. Regularly	<0.005 *
					Occasionally vs. Regularly	<0.005 *
POMA—Tinetti	132 (68.1)	47 (24.2)	15 (7.7)	<0.005 *	Never vs. Occasionally	<0.005 *
					Never vs. Regularly	<0.005 *
					Occasionally vs. Regularly	<0.005 *
Turn Test 360°	98 (50.5)	58 (29.9)	38 (19.6)	<0.005 *	Never vs. Occasionally	0.001 *
					Never vs. Regularly	<0.005 *
					Occasionally vs. Regularly	0.041 *
FGA	35 (18.0)	74 (38.2)	85 (43.8)	<0.005 *	Never vs. Occasionally	<0.005 *
					Never vs. Regularly	<0.005 *
					Occasionally vs. Regularly	0.383
TIS	107 (55.2)	63 (32.4)	24 (12.4)	<0.005 *	Never vs. Occasionally	0.001 *
					Never vs. Regularly	<0.005 *
					Occasionally vs. Regularly	<0.005 *
PASSP	100 (51.5)	61 (31.5)	33 (17.0)	<0.005 *	Never vs. Occasionally	0.002 *
					Never vs. Regularly	<0.005 *
					Occasionally vs. Regularly	0.004 *
TUG	43 (22.2)	82 (42.2)	69 (35.6)	0.002 *	Never vs. Occasionally	<0.005 *
					Never vs. Regularly	0.014 *
					Occasionally vs. Regularly	0.290
STS	48 (24.7)	77 (39.7)	69 (35.6)	0.031 *	Never vs. Occasionally	0.009 *
					Never vs. Regularly	0.052
					Occasionally vs. Regularly	0.508

The table shows that the most regularly used balance assessment tools were the SLS, BBS, FGA, and TUG. In contrast, tools like the POMA—Tinetti, MBEST, and FES-I were rarely used. ABC: Activity-Specific Balance Confidence Scale; BBS: Berg Balance Scale; FES-I: Fall Efficacy Scale International; DGI: Dynamic Gait Index; FRT: Functional Reach Test; SLS: Single-Leg Stance; FSST: Four-Step Square Test; Y-Balance: Y-Balance Test Lower Quarter; MBEST: Mini Balance Evaluation Systems Test; POMA—Tinetti: Performance-Oriented Mobility Assessment; FGA: Functional Gait Assessment; TIS: Trunk Impairment Scale; PASSP: Postural Assessment Scale for Stroke Patients; TUG: Timed Up and Go; STS: Five Times Sit-to-Stand. Note: Significant differences were examined using Chi-square tests. * Significant difference at α = 0.05.

**Table 3:** Effect of participants’ area of practice, degree, and practicing experience on tool preferences (n = 194).

Scale	Area of Practice				Degree				Practicing Experience (Years)			
	Attribute	*n* (%)	*p*	ES	Attribute	*n* (%)	*p*	ES	Attribute	*n* (%)	*p*	ES
ABC	No significant preference detected											
BBS	Neuro	38 (48.1)	0.007	0.36	Master’s	25 (59.5)	0.001	0.57	1–5	39 (43.8)	0.033	0.28
	Pediatric	12 (57.1)	0.028	0.58	Doctoral	11 (64.7)	0.011	0.73	≥11	19 (61.3)	0.004	0.60
FES-I	No significant preference detected											
DGI	No significant preference detected											
FRT	No significant preference detected											
SLS	Neuro	43 (54.4)	<0.005	0.53	Bachelor’s	71 (55.9)	<0.005	0.50	1–5	53 (59.6)	<0.005	0.61
	Sports	17 (53.1)	0.033	0.46								
	Ortho	20 (71.4)	<0.005	0.81	Master’s	22 (52.4)	<0.005	0.62	≥11	19 (61.3)	0.003	0.61
	Pediatric	15 (71.4)	0.001	0.82								
FSST	No significant preference detected											
Y-Balance	No significant preference detected											
MBEST	No significant preference detected											
POMA—Tinetti	No significant preference detected											
Turn Test 360°	No significant preference detected											
FGA	Neuro	35 (44.3)	0.003	0.39	Bachelor’s	56 (44.1)	0.001	0.33	1–5	44 (49.4)	<0.005	0.41
	Ortho	16 (57.1)	0.025	0.51								
TIS	No significant preference detected											
PASSP	No significant preference detected											
TUG	Neuro	34 (43.0)	0.001	0.41	Master’s	19 (45.2)	0.005	0.51	≥11	14 (45.2)	0.020	0.50
STS	No significant preference detected											

The table indicates significant associations between physical therapists’ area of practice, degree, and experience and their preference for specific tools. Particularly, pediatric and orthopedic practitioners, master’s and doctoral degree holders, and those with ≥11 years of experience showed stronger preferences for BBS and SLS. FGA and TUG also showed significant variation with these factors. ES: Cohen’s ω effect size; ABC: Activity-Specific Balance Confidence Scale; BBS: Berg Balance Scale; FES-I: Fall Efficacy Scale International; DGI: Dynamic Gait Index; FRT: Functional Reach Test; SLS: Single-Leg Stance; FSST: Four-Step Square Test; Y-Balance: Y-Balance Test Lower Quarter; MBEST: Mini Balance Evaluation Systems Test; POMA—Tinetti: Performance-Oriented Mobility Assessment; FGA: Functional Gait Assessment; TIS: Trunk Impairment Scale; PASSP: Postural Assessment Scale for Stroke Patients; TUG: Timed Up and Go; STS: Five Times Sit-to-Stand. Note: *p*-values were calculated using Chi-square tests.

**Table 4:** Effects of the age range of the participants assessed and treated, facility type, and working environment on tool preferences (n = 194).

Scale	Age Range of Patients Assessed and Treated				Facility Type				Working Environment			
	Attribute	*n* (%)	*p*	ES	Attribute	*n* (%)	*p*	ES	Attribute	*n* (%)	*p*	ES
ABC	No significant preference detected											
BBS	Children and adolescents	19 (51.4)	0.032	0.43	Public	48 (50.5)	<0.005	0.43	Multidisciplinary	33 (48.5)	0.001	0.47
					University	8 (72.7)	0.020	0.84				
FES-I	No significant preference detected											
DGI	No significant preference detected											
FRT	No significant preference detected											
SLS	Children and adolescents	23 (62.2)	<0.005	0.64	Public	53 (55.8)	<0.005	0.52	Individually	40 (53.3)	<0.005	0.47
	Early-aged adults	37 (52.9)	<0.005	0.47					With other PTs	28 (54.9)	0.001	0.51
	Middle-aged adults	34 (56.7)	<0.005	0.57	Private	46 (52.3)	<0.005	0.49	Multidisciplinary	38 (55.9)	<0.005	0.58
FSST	No significant preference detected											
Y-Balance	No significant preference detected											
MBEST	No significant preference detected											
POMA—Tinetti	No significant preference detected											
Turn Test 360°	No significant preference detected											
FGA	Middle-aged adults	28 (46.7)	0.008	0.40	Public	42 (44.2)	0.005	0.34	With other PTs	22 (43.1)	0.012	0.42
					Private	38 (43.2)	0.009	0.33	Multidisciplinary	34 (50.0)	0.002	0.43
TIS	No significant preference detected											
PASSP	No significant preference detected											
TUG	Middle-aged adults	27 (45.0)	0.019	0.36	No significant preference detected							
STS	No significant preference detected											

The table indicates that tool preferences varied significantly based on patient age group, facility type, and working environment. The SLS showed a consistent significant preference across all three categories. The BBS and FGA also displayed significant variation, particularly among those working in public or multidisciplinary settings. ES: Cohen’s ω effect size; ABC: Activity-Specific Balance Confidence Scale; BBS: Berg Balance Scale; FES-I: Fall Efficacy Scale International; DGI: Dynamic Gait Index; FRT: Functional Reach Test; SLS: Single-Leg Stance; FSST: Four-Step Square Test; Y-Balance: Y-Balance Test Lower Quarter; MBEST: Mini Balance Evaluation Systems Test; POMA—Tinetti: Performance-Oriented Mobility Assessment; FGA: Functional Gait Assessment; TIS: Trunk Impairment Scale; PASSP: Postural Assessment Scale for Stroke Patients; TUG: Timed Up and Go; STS: Five Times Sit-to-Stand. Note: *p*-values were calculated using Chi-square tests.

## Data Availability

The data presented in this study are available on request from the corresponding author due to ethical reasons.

## Abbreviations

The following abbreviations are used in this manuscript:

ABC	Activity-Specific Balance Confidence Scale
BBS	Berg Balance Scale
FES-I	Falls Efficacy Scale International
DGI	Dynamic Gait Index
FRT	Functional Reach Test
FSST	Four-Step Square Test
FGA	Functional Gait Assessment
PASS-P	Postural Assessment Scale for Stroke Patients
POMA—Tinetti	Performance-Oriented Mobility Assessment
MBEST	Mini Balance Evaluation Systems Test
SLS	Single-Leg Stance
STS	Five Times Sit-to-Stand
TIS	Trunk Impairment Scale
TUG	Timed Up and Go
Y-Balance	Y-Balance Test Lower Quarter
